# Resting State Functional Connectivity between Dorsal Attentional Network and Right Inferior Frontal Gyrus in Concussed and Control Adolescents

**DOI:** 10.3390/jcm11092293

**Published:** 2022-04-20

**Authors:** Stephen J. Suss, Anna Manelis, Joao Paulo Lima Santos, Cynthia L. Holland, Richelle S. Stiffler, Hannah B. Bitzer, Sarrah Mailliard, Madelyn Shaffer, Kaitlin Caviston, Michael W. Collins, Mary L. Phillips, Anthony P. Kontos, Amelia Versace

**Affiliations:** 1 Department of Psychiatry, University of Pittsburgh, Pittsburgh, PA 15260, USA; susssj2@upmc.edu (S.J.S.); santosjp@upmc.edu (J.P.L.S.); stifflerrs@upmc.edu (R.S.S.); sarrah.mailliard@gmail.com (S.M.); phillipsml@upmc.edu (M.L.P.); versacea@upmc.edu (A.V.); 2 UPMC Sports Medicine Concussion Program, Department of Orthopaedic Surgery, University of Pittsburgh, Pittsburgh, PA 15260, USA; clh197@pitt.edu (C.L.H.); hbb11@pitt.edu (H.B.B.); mas695@pitt.edu (M.S.); kcaviston@pitt.edu (K.C.); collinsmw@upmc.edu (M.W.C.); akontos@pitt.edu (A.P.K.); 3 Magnetic Resonance Research Center, Department of Radiology, University of Pittsburgh, Pittsburgh, PA 15260, USA

**Keywords:** concussion, adolescents, resting state functional connectivity, dorsal attention network, inferior frontal gyrus

## Abstract

Concussion among adolescents continues to be a public health concern. Yet, the differences in brain function between adolescents with a recent concussion and adolescents with no history of concussion are not well understood. Although resting state functional magnetic resonance imaging (fMRI) can be a useful tool in examining these differences, few studies have used this technique to examine concussion in adolescents. Here, we investigate the differences in the resting state functional connectivity of 52 adolescents, 38 with a concussion in the previous 10 days (mean age = 15.6; female = 36.8%), and 14 controls with no concussion history (mean age = 15.1; female = 57.1%). Independent component analysis and dual regression revealed that control adolescents had significantly greater functional connectivity between the dorsal attention network (DAN) and right inferior frontal gyrus (RIFG) compared to concussed adolescents (*p*-corrected < 0.001). Specifically, there was a positive DAN-RIFG connectivity in control, but not concussed, adolescents. Our findings indicate that concussion is associated with disrupted DAN-RIFG connectivity, which may reflect a general, nonspecific response to injury.

## 1. Introduction

Adolescent concussion is a growing public health concern, with upwards of 1.9 million incidents occurring each year [1,2]. Emerging evidence indicates that adolescents often experience more severe post-concussion symptoms and longer recovery times than adults [3,4] and younger children [5]. The acute (<3 days post injury) and subsequent subacute phases of concussion are often characterized by headaches, fatigue, dizziness, nausea, sleep disturbance, psychological symptoms, and cognitive dysfunction [6,7]. These symptoms generally last for up to four weeks [8] and can interfere with adolescents’ ability to participate in school, sports, and social activities [9,10,11].

Understanding the neurobiological underpinnings of adolescent concussion is important for predicting patients’ recovery trajectory, informing future treatments, and improving outcomes. Resting state functional MRI (fMRI) is a brain imaging technique that examines brain function in the absence of external stimuli. Resting state functional connectivity refers to synchronous fluctuations in blood oxygenation level-dependent (BOLD) fMRI signal implying functional relationships between distinct brain regions at rest [12,13,14]. Several resting state networks, such as the Default Mode (DMN), the executive function (EFN), ventral attention (VAN), and dorsal attention (DAN) networks, have consistently been identified as disrupted in a number of clinical populations [12,13,15]. Resting state fMRI provides a unique opportunity to examine functional connectivity in the brain of concussed adolescents without necessitating their engagement in cognitive tasks that might exacerbate fatigue, dizziness, and other symptoms during the subacute post-concussion period.

To our knowledge, only two previous fMRI studies with relatively small sample sizes have compared resting state functional connectivity between concussed and non-concussed adolescents. The first study investigated concussed adolescents (*n* = 12) outside of the subacute post-concussion period (up to two months past injury) versus non-concussed controls (*n* = 10) and revealed increased functional connectivity in the executive function (EFN) and ventral attention (VAN) networks, as well as mixed findings in the Default Mode network (DMN), in concussed compared to non-concussed adolescents [16]. The second study compared adolescent male hockey players in the subacute post-concussion period (*n* = 16, past 7 days post-concussion) with non-concussed adolescent male hockey players (*n* = 12) and revealed hyperconnectivity in posterior regions and hypoconnectivity in anterior regions, albeit in different regions than the prior study [16,17]. In addition, they found that the functional connectivity between the DMN and the right middle temporal and precentral gyrus positively correlated with visual memory, as measured by the Immediate Post-concussion Assessment and Cognitive Test (ImPACT), whereas functional connectivity between DMN and the regions in the inferior parietal lobe, middle cingulate, and postcentral and lingual gyrus positively correlated with ImPACT verbal memory scores [17].

Considering that the generalizability of these studies is limited by their small sample sizes, use of varying posts-concussion periods, participant selection criteria, or small number of networks of interest, we aimed to address these shortcomings by examining resting-state functional connectivity using a whole brain approach and a larger sample of concussed male and female adolescents who sustained a concussion within 10 days. We hypothesized that concussed adolescents would differ from never-concussed (control) adolescents in functional connectivity between the DMN and the prefrontal cortical regions. We also hypothesized that connectivity in the regions showing significant differences between concussed and control adolescents would positively correlate with ImPACT scores, post-concussion symptom severity, and self-reported vestibular-ocular-motor symptoms.

## 2. Materials and Methods

### 2.1. Participants

The study was approved by the University of Pittsburgh Institutional Review Board protocol number STUDY19030360. Informed consent was obtained from all adolescents involved in the study and assent was obtained from their parents. Study recruitment occurred through an ongoing study, namely the Investigating Concussion in Adolescents at Risk for Emotion dysregulation (iCARE) study. The concussed group consisted of adolescents aged 12–17 years who sought care for concussion symptoms at the UPMC Concussion Clinic and received a formal diagnosis of concussion per current consensus criteria [8] within 1–10 days. Adolescents who lost consciousness for more than 5 min were excluded. Control adolescents with no past or current history of concussion were recruited through a local recruitment website Pitt + Me and age- and sex-matched to concussed adolescents. Exclusion criteria for all adolescents included neurologic, neurodevelopmental, or systemic medical disease, orthopedic injury within the past month, personal history of psychotic spectrum disorders (i.e., schizophrenia, bipolar disorder), current (i.e., within the past three months) alcohol or illicit substance use/dependency, left/mixed handedness, IQ below 70, MRI contraindications, and intoxication or presence of illicit drugs in a urine test on the day of the scan. The International Neuropsychiatric Interview for children and adolescents (MINI-KID) was used to ascertain the absence of psychiatric disorders [18] and/or current use of psychotropic medications. In cases of equivocal symptom reporting, results of the MINI screening were reviewed by the principal investigator.

A total of 96 adolescents aged 12–17 (65 concussed and 31 controls) were enrolled and participated in a 6.5-min resting-state scan. Wakefulness inside the scanner was assessed using a post-scan self-report questionnaire, where participants were asked for what percent of time during the scan they were thinking or “doing” a certain activity (e.g., thinking about the past, thinking about the presenting, daydreaming, etc.). They would also indicate what percent of the scan they believed to have slept through; those reporting to have slept for >50% of the scan were excluded from analysis. Three control adolescents (9.7%) were removed due to endorsing falling asleep during most of the resting-state scan. No concussed adolescents endorsed sleeping during the scan. However, post-scan self-reported sleep data was missing for 19 concussed participants (29.2%), and they were removed from analysis to ensure only resting state data for awake participants was used. Additionally, 8 concussed adolescents (12.3%) and 14 control adolescents (45.2%) were excluded due to excessive head motion (>2 mm). Overall, 52/96 (54.2%) participants including 38 concussed adolescents (mean [SD] days since injury = 6.6 [2.2]) and 14 control adolescents (see Table 1 for clinical characteristics and demographics) were included in the analyses. For more information, see Table S1.

One concussed adolescent met criteria for generalized anxiety disorder, one concussed adolescent met criteria for both generalized anxiety disorder and ADHD, one concussed adolescent met criteria for major depressive and separation anxiety disorders, one concussed adolescent was on amitriptyline despite not meeting full criteria for any psychiatric disorder, and three individuals (two concussed and one control) met criteria for ADHD alone. To ensure that our results do not depend on the presence of these disorders or the use of psychotropic medication, we repeated the analyses without the seven individuals described above.

### 2.2. Clinical Assessments of Concussion

Three commonly used measures of concussion were employed in this study, including: ImPACT [19], Post-Concussion Symptom Scale (PCSS) [20], and Vestibular/Ocular-Motor Screening (VOMS) [21]. Higher scores on all three assessments indicates greater severity. The ImPACT includes six cognitive assessment modules that comprise four composite scores of verbal and visual memory, visual motor processing speed, and reaction time. The PCSS, which is embedded in the ImPACT tool, measures the severity of 22 commonly reported symptoms such as headaches, dizziness, and nausea that comprise four main symptom factors: cognitive-migraine-fatigue, affective, somatic, and sleep [22]. The VOMS tool includes seven items that assess symptom provocation following performance of smooth pursuits, horizontal and vertical saccades, near point convergence (NPC), horizontal and vertical vestibular ocular reflex (VOR), and visual motion sensitivity (VMS); as well as an average measure (3 trials) of NPC distance (in cm). All concussion measures were administered by clinical members or the research team at patients’ first clinical visit for their concussion. Relevant demographics including age, sex, and medical and injury history were collected. History of prior concussions, migraine, and motion sickness, as well as the mechanism of current injury were also collected (Table 1).

### 2.3. Neuroimaging Data Acquisition

Neuroimaging data were collected using a 3T Siemens Magnet Prisma MR scanner using a 32-channel head coil. A high-resolution structural image was acquired using the MPRAGE sequence (voxel size = 1 × 1 × 1 mm^3^, TR = 2400 ms, FOV = 256 mm, flip angle = 8°, 176 slices). A 6.5-min resting-state scan was acquired using an echo-planar image (EPI) sequence in the anterior to posterior direction (voxel size = 2 × 2 × 2 mm^3^, TR = 800 ms, TE = 30.00 ms, FOV = 210 mm, flip angle = 52°, 72 slices, 500 volumes, multiband acceleration factor = 8). During the resting state sequence, participants were instructed to lay still and stay awake with eyes open. We also collected two spin echo images with the anterior-to-posterior and posterior-to-anterior phase encoding directions (voxel size = 2 × 2 × 2 mm^3^, TR = 8000ms, TE = 66.00 ms, FOV = 210 mm, flip angle = 90°, 72 slices).

### 2.4. fMRI Data Preprocessing

DICOM files were converted to NIFTI using the dcm2niix (version v1.0.20180826 BETA GCC5.4.0, Creator: Chris Rorden) [23] tool. The optiBET script (Creator: Evan Lutkenhoff, https://montilab.psych.ucla.edu/fmri-wiki/optibet, accessed on 26 March 2022) [24] was used to remove non-brain tissues. The two spin-echo images were fed into topup (https://fsl.fmrib.ox.ac.uk/fsl/fslwiki/topup, accessed on 26 March 2022) in FSL6.0.3 (www.fmrib.ox.ac.uk/fsl, accessed on 26 March 2022) to reduce susceptibility distortions in the BOLD images. Motion correction was performed using MCFLIRT [25] and spatial smoothing with a Gaussian kernel of full-width at half-maximum = 6 mm was applied. In order to transform BOLD images to MNI space, BOLD images were first registered to the high-resolution structural (MPRAGE) images using FLIRT (FMRIB’s Linear Image Registration Tool, version 6.0, https://fsl.fmrib.ox.ac.uk/fsl/fslwiki/FLIRT, accessed on 26 March 2022) [25,26] with Boundary-Based Registration (BBR); the high-resolution images were registered to the MNI152 T1-2 mm template using FNIRT (FMRIB’s Non-linear Image Registration Tool, https://fsl.fmrib.ox.ac.uk/fsl/fslwiki/FNIRT, accessed on 26 March 2022) [27] with nine degrees of freedom (DOF), and the two resulting transformations were concatenated and applied to the original BOLD. The quality of transformation was inspected for every subject.

ICA-AROMA (version v0.3-beta, https://github.com/maartenmennes/ICA-AROMA, accessed on 26 March 2022) [28] was applied on preprocessed and normalized BOLD images to remove motion artifacts. The fsl_anat script (https://fsl.fmrib.ox.ac.uk/fsl/fslwiki/fsl_anat, accessed on 26 March 2022) was used to segment high-resolution structural images to white matter, grey matter, and cerebral-spinal fluid (CSF) masks. The white matter and CSF masks were then co-registered with functional images. Their time courses were then extracted from the preprocessed functional data. The white matter and CSF time courses and first 5 TR were then regressed out from the preprocessed BOLD images. Subsequently, a band-pass filter of 0.01–0.08 Hz (Gaussian-weighted least-squares straight line fitting, with sigma1 = 7.8125 (0.08 Hz) and sigma2 = 62.5 (0.01 Hz)) was applied.

### 2.5. fMRI Data Analysis

Preprocessed resting-state data from all participants served as input for conducting group independent component analysis (ICA) with a 30-component solution utilizing MELODIC software (version 3.15, Creator: Christian F. Beckmann, https://fsl.fmrib.ox.ac.uk/fsl/fslwiki/MELODIC, accessed on 26 March 2022) in FSL 6.0.3. Using this analysis, large-scale networks of functionally connected regions were determined across all participants [29]. Thresholded component maps (or networks) were visually inspected and cross-correlated with the well characterized Yeo’s (2011) 7-network maps using FSL’s fslcc tool (https://fsl.fmrib.ox.ac.uk/fsl/fslwiki/Fslutils, accessed on 26 March 2022) to understand the spatial overlap between the networks in our study and those observed in an independent sample of 1000 control subjects in Yeo’s study [30].

The between-group differences (2 levels: concussed and control) were examined using dual-regression in FSL [14,31]. This tool uses group-ICA spatial maps in a linear fit model to identify subject-specific temporal dynamics and associated spatial maps [14,32]. Thresholded maps that exceeded a 0.25 spatial correlation with Yeo’s networks were combined to create a 4D volume that served as input for dual regression. Within dual-regression, we used the randomize tool with 10,000 permutations and TFCE to determine significant differences between groups [32]. Given that the sex and age of adolescents could affect their functional connectivity, both of these variables were included in our model as covariates [33]. To account for the number of comparisons, *p*-values were Bonferroni corrected across all maps included into the analyses and all contrasts computed for each map.

The parameter estimates were extracted from the brain regions showing significant group differences and submitted to linear regressions that determined whether aberrant functional connectivity in concussed adolescents was associated with the IMPACT, PCSS, or VOMS scores.

### 2.6. Exploratory Analyses

To assess if our functional connectivity findings depended on age or sex, we conducted two analyses of variance (ANOVA): one for age-by-group interaction and the other one for sex-by-group interaction. We also compared functional connectivity findings between concussed adolescents with vs. without history of motion sickness, migraines, or prior concussion, with respectively tailed *t*-tests.

## 3. Results

### 3.1. Demographic and Clinical Data Analyses

There were no significant differences between concussed and control adolescents in age, sex, race composition, or IQ (Table 1).

### 3.2. Neuroimaging Data Analysis

**Figure 1:**
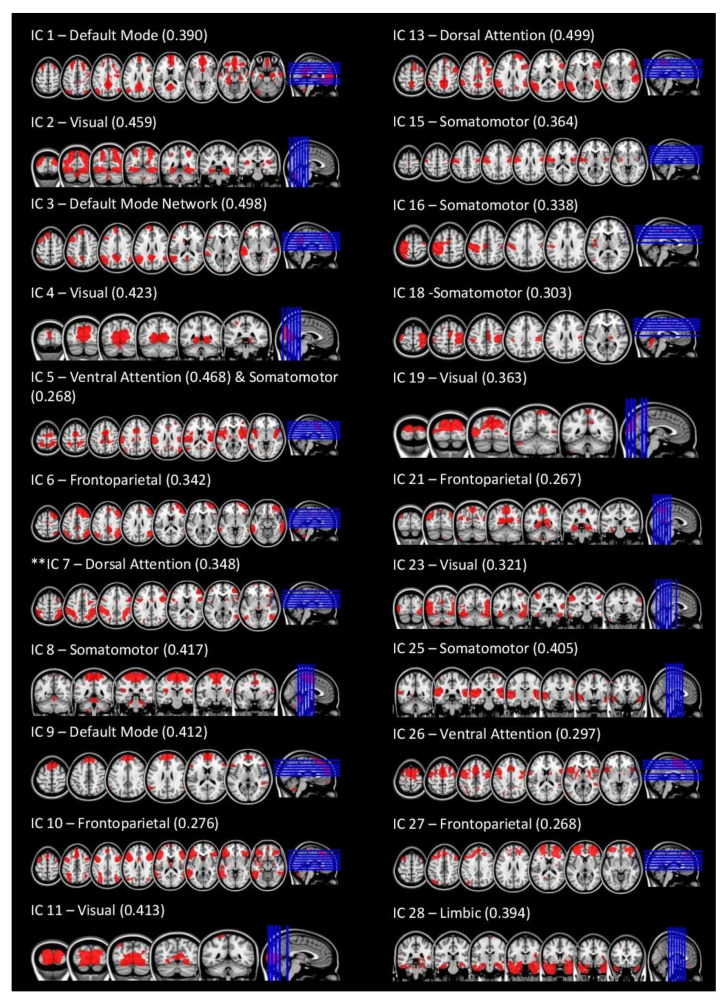
Results for Group ICA. Independent components (IC) were labeled and organized based on their cross-correlations with Yeo’s (2011) networks. Above is each IC, the associate Yeo network(s), and the cross-correlation coefficient between the two. ** Denotes Network of interest.

The cross-correlation analysis revealed that 22 independent components cross-correlated with Yeo’s 7-network solution maps above r = 0.25 (Figure 1). Each of these components was labeled according to the Yeo’s network(s) that cross-correlated with that component. The remaining eight components were removed from the analyses. To account for the multiple tests that included using 2 contracts (concussed > control and control > concussed) for each of the 22 networks, the *p*-values were Bonferroni corrected accordingly (0.05/22 × 2 = 0.0011).

**Figure 2:**
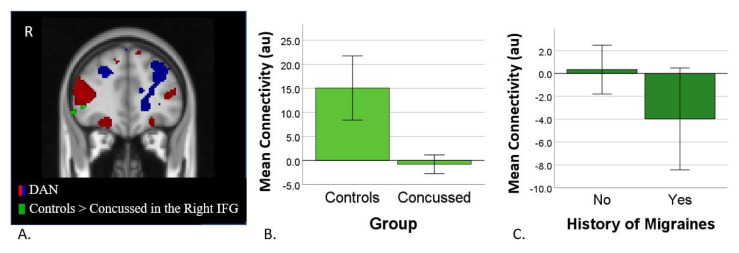
(**A**) Independent component (IC 7, red & blue) and region (RIFG) in which there is a significant difference in connectivity between control and concussed participants (green). (**B**) Bar graph illustrating mean parameter estimates in arbitrary units for the DAN-RIFG connectivity in control and concussed adolescents. (**C**) Bar graph illustrating mean DAN-RIFG connectivity for concussed participants with a history of migraine vs. those without.

Dual regression analysis revealed that functional connectivity between the Dorsal Attention Network (DAN) (IC7) and the right inferior frontal gyrus (RIFG) was significantly greater in control vs. concussed adolescents (t-max = 6.2, nvox = 34, *p* = 0.0008, MNI coordinates (x = 22, y = 81, z = 36); Figure 2A). Specifically, control adolescents demonstrated positive functional connectivity between the DAN and RIFG (t(14) = 4.9, *p* < 0.001), suggesting that when activation in the DAN increases, it also increases in the RIFG. In contrast, concussed adolescents showed no connectivity between the two regions (t(38) = −0.8, *p* = 0.413; Figure 2B). In concussed adolescents, the measures of DAN-RIFG connectivity did not correlate with the IMPACT, PCSS, or VOMS scores (see Table S2).

The contrast between concussed vs. control adolescents for DAN-RIFG connectivity remained significant (t(45) = −4.6, *p* = 0.0004) even after removing participants taking psychotropic medications or diagnosed with anxiety disorders or ADHD (*n* = 7). Again, control adolescents demonstrated a positive connectivity between the DAN and RIFG (t(13) = 4.8, *p* < 0.001). Concussed adolescents showed no significant DAN-RIFG connectivity (t(32) = 0.0, *p* = 0.977) and no significant relationship between DAN-RIFG connectivity and the IMPACT, PCSS, or VOMS scores (see Table S3).

### 3.3. Exploratory Analyses

Our exploratory analyses revealed that the functional connectivity patterns between the DAN and RIFG were unrelated to participants’ age and sex. No significant differences in DAN-RIFG connectivity were found between concussed adolescents with a history of motion sickness or concussion vs. those without such histories.

DAN-RIFG functional connectivity was significantly greater in concussed adolescents without a self-reported history of migraines vs. those with a history of migraines (t(38) = 2.1, *p* = 0.047). Although connectivity in the former group was not statistically significant from zero (t(10)=0.3, *p* = 0.75), concussed adolescents with a history of migraines showed a trend toward a negative relationship between the DAN and RIFG (t(28) = −2.0, *p* = 0.075; Figure 2C). Of note, when concussed adolescents with a history of psychiatric disorders or psychotropic medication use were removed (*n* = 7), the differences between concussed adolescents with and without history of migraines became non-significant.

## 4. Discussion

In this study, we examined the differences in resting state functional connectivity between concussed adolescents and age- and gender-matched controls with no history of concussion. The key finding supported our first hypothesis and indicated that concussed adolescents, compared to controls, had significantly reduced functional connectivity between the DAN and the RIFG. Specifically, controls demonstrated a significant positive DAN-RIFG functional connectivity, whereas DAN-RIFG connectivity in concussed adolescents was not significantly different from zero. In contrast to our secondary hypothesis, DAN-RIFG functional connectivity was not associated with scores on the ImPACT, PCSS, or VOMS for concussed adolescents. Although we did find that lower DAN-RIFG functional connectivity was associated with a history of migraine within the concussed adolescent group, this relationship became not significant after we removed the individuals with psychiatric disorders from the analyses. The latter could be explained by the collinearity between the presence of psychiatric disorders and migraines as well as by the reduction in sample size.

The DAN is primarily involved in goal-directed attention and the top-down selection of sensory content by demonstrating sustained activation when individuals are engaged in focused attention [34,35]. Considering that the RIFG plays an important role in shifting attention [36,37], disruptions in DAN-RIFG connectivity may potentially affect an individual’s ability to orient and subsequently maintain focus of attention during tasks. Given that post-concussion symptoms were not significantly related to the magnitude of the DAN-RIFG connectivity, we propose that the reduction in this connectivity may reflect a global non-specific brain response to concussion that occurs during the subacute phase of post-concussion recovery.

Our results were inconsistent with the findings of the few previous resting state studies that found alterations in the EFN, VAN, and DMN between concussed and control adolescents [16,17]. Unlike Murdaugh et al. (2018), we employed a whole-brain approach that allowed us to investigate the effect of concussion on multiple resting state networks. Considering that this approach required us to account for multiple tests, only large effects survived correction for multiple comparisons. Nonetheless, we cannot exclude the possibility that a functional deficit in a resting state network may become evident in different stages of post-concussion recovery. For example, the DAN network might be affected directly by the injury and thus precede alterations in other networks, whereas the EFN, VAN, and DMN disruption could occur later due to a ‘secondary’ response of concussion in reaction to the primary damage (e.g., disruption in the DAN). This could explain why our findings varied from Borich et al. (2015), whose sample consisted of adolescents over a much longer concussion recovery period.

It is important to note that unlike the prior resting state study [17], we observed no correlation between functional connectivity and post-concussion symptoms. One potential explanation for the lack of the effect is that a disruption in DAN-RIFG connectivity may be a “all or none” response to injury that reflects global non-specific post-concussion changes. Given that there were no significant differences in DAN-RIFG connectivity between concussed adolescents with and without prior history of concussion, we believe that after recovery, the DAN-RIFG connectivity likely returns to baseline. Further investigation is needed to better understand how DAN-RIFG contributes to the presence or absence of post-concussion symptoms and how this connectivity might be related to recovery trajectory.

Although marginal and only relevant to our full sample of concussed adolescents, our findings of significant differences in DAN-RIFG connectivity between adolescents with a history of migraine and those without is a possible area for further investigation. While the DAN-RIFG connectivity did not differ from zero in both concussed adolescents with and without such a history, there was a trend toward a negative association between these regions in concussed adolescents with a history of migraines. Prior resting state studies demonstrated that adolescents who experience migraines have significantly altered patterns of connectivity when compared to controls as well as younger children [38] and adults [39] suffering from migraines. Importantly, functional connectivity in adolescents with migraines is dependent on age and time between the last migraine attack and scan [38]. Considering that symptoms of a migraine may overlap with those experienced during the earlier stages of concussion (e.g., headaches, dizziness, fogginess, nausea), this finding might indicate that adolescents with migraines are more vulnerable to DAN-RIFG connectivity disruption due to concussion than adolescents without premorbid migraines. Unfortunately, the small sample of concussed adolescents with migraines (*n* = 10) and the lack of information about migraine history in the control adolescents prevent us from drawing definitive conclusions regarding the relationship between the DAN-RIFG functional connectivity in the subacute post-concussion period and migraine history. Future studies with larger samples are needed to better understand the relationship between concussion and migraine in relation to DAN-RIFG connectivity.

There were several limitations in this study. First, a lack of premorbid resting state fMRI data precluded us from assessing individual neuroimaging changes due to concussion in the study sample. Second, we had no data on migraine or motion sickness history for controls, so we could not determine whether DAN-RIFG functional connectivity was reduced in control adolescents with these histories, as we could for concussed adolescents. Furthermore, although we used a larger sample size than prior studies, we had relatively few controls and only a small number of participants with migraine history. Future studies should examine larger sample sizes of adolescents with preexisting migraines and other somatic symptoms to monitor their potential impact on concussive injury and outcomes. Additionally, these studies should examine a variety of networks at different stages of recovery to better characterize brain activity and its relation to symptom severity over the course of injury.

## 5. Conclusions

Our study demonstrated that control adolescents without a history of concussion have positive functional connectivity between the DAN and RIFG, whereas concussed adolescents within 10 days of injury show a disconnection between the DAN and RIFG. The degree of dysconnectivity between these regions was not associated with measures of concussion symptoms, suggesting that the disruption of DAN-RIFG connectivity reflects a general, nonspecific response to injury. This finding may point to an important biomarker, in which the presences or absence of DAN-RIFG connectivity could be a clear measure of concussive injury in adolescences. It would be important to monitor whether DAN-RIFG dysconnectivity persist in adolescents whose concussion symptoms do not resolve and where targeted interventions utilizing neuroplasticity might be used to restore connectivity. A history of migraine may further disrupt DAN-RIFG connectivity in concussed adolescents, but further investigation is needed to understand the contribution of a history of migraine and history of psychiatric disorders, both independently and in combination, on resting state functional connectivity in concussed adolescents.

## Figures and Tables

**Table 1:** Subject demographics and clinical characteristics.

Demographics	Concussed N = 38	Controls N = 14	*t*-Test/Chi-Square Concussed vs. Control
Age, mean [SD]	15.6 [1.6]	15.1 [1.7]	t(52) = 1.1, *p* = 0.293
IQ, mean [SD]	105.9 [7.9]	107.1 [7.9]	t(52) = 0.4, *p* = 0.646
Sex			
Male (%)	24 (63.2%)	6 (42.9%)	χ^2^ = 1.7, *p* = 0.189
Female (%)	14 (36.8%)	8 (57.1%)	
Race			
White (%)	32 (84.2%)	9 (64.3%)	χ^2^ = 3.6, *p* = 0.116
Black (%)	5 (13.2%S)	5 (35.7%)	
More than one (%)	1 (2.6%)	0 (0.0%)	
Ethnicity			
Non-Hispanic (%)	36 (94.7%)	12 (85.7%)	χ^2^ = 1.2, *p* = 0.556
Hispanic	1 (2.6%)	1 (7.1%)	
Unknown	1 (2.6%)	1 (7.1%)	
ImPACT composite scores			
Verbal memory (%), mean [SD]	77.2 [13.1]	-	
Visual memory (%), mean [SD]	68.5 [14.0]	-	
Visual motor processing speed, mean [SD]	33.9 [8.3]	-	
Reaction time (sec), mean [SD]	0.69 [0.1]	-	
Concussion Symptoms			
Affective factor, mean [SD]	2.4 [2.9]	-	
Somatic factor, mean [SD]	0.5 [1.0]	-	
Sleep factor, mean [SD]	1.7 [2.3]	-	
Cognitive-migraine-fatigue factor, mean [SD]	17.3 [12.2]	-	
VOMS total symptom score, mean [SD]	51.7 [41.8]	-	
History of prior concussion			
Yes (%)	13 (34.2%)	0 (0%)	
No(%)	25 (65.8%)	0 (0%)	
History of migraines			
Yes (%)	10 (26.3%)	-	
No(%)	28 (73.7%)	-	
History of motion sickness			
Yes (%)	6 (15.8%)	-	
No (%)	32 (84.2%)	-	
Mechanism of Injury			
Sport-related	33 (87%)	-	
Non-sport (MVC, falls, assaults)	5 (13%)	-	

## Data Availability

The data may be available upon a reasonable request.

## Supplementary Materials

The following supporting information can be downloaded at: https://www.mdpi.com/article/10.3390/jcm11092293/s1, Table S1: Demographics for included vs. excluded participants; Table S2: The effect of ImPACT scores, PCSS symptom factors, VOMS, and medical history on RIFG-DAN connectivity in the full sample of concussed adolescents (*n* = 38); Table S3: The effect of ImPACT scores, PCSS symptom factors, VOMS, and medical history on RIFG-DAN connectivity in the sample of concussed adolescents without a history of psychiatric disorders or using psychotropic medications (*n* = 31).
