# Supplementary material for: Resting State Functional Connectivity between Dorsal Attentional Network and Right Inferior Frontal Gyrus in Concussed and Control Adolescents
Source: J Clin Med. 2022 Apr 20;11(9):2293. doi: 10.3390/jcm11092293 (PMC9100070; doi:10.3390/jcm11092293)
Supplement: Supplementary file 1 [file jcm-11-02293-s001.zip › jcm-1678849-supplementary.pdf]

Supplemental Tables

Table S1. Demographics for included vs. excluded participants.

Demographics	**Included (52)**	**Excluded (44)**	**t or χ^2**	***p*-Value**
Age, mean [SD]	15.5 [1.6]	15.2 [1.4]	0.8	0.429
IQ	106.4 [7.9]	106.1 [8.9]†	0.2	0.857
**Sex**				
Male (%)	30 (57.7%)	25 (56.8%)	0.0	0.931
Female (%)	22 (42.3%)	19 (43.2%)		
**Race**				
White (%)	41 (78.8%)	38 (86.4%)	5.3	0.262
Black or AA (%)	10 (19.2%)	3 (6.8%)		
Asian (%)	0 (0.0%)	1 (2.3%)		
More than one (%)	1 (1.9%)	1 (2.3%)		
Unknown (%)	0 (0.0%)	1 (2.3%)		
**Ethnicity**				
Non-Hispanic (%)	48 (92.3%)	40 (90.9%)	0.1	0.970
Hispanic	2 (3.8%)	2 (4.5%)		
Unknown	2 (3.8%)	2 (4.5%)		

Note: †IQ data is missing for two subjects † ı

Table S2. The effect of ImPACT scores, PCSS symptom factors, VOMS, and medical history on RIFG-DAN connectivity in the full sample of concussed adolescents (*n* = 38).

Variable	**F[1, 36] or t(37)**	***p*-Value**
**ImPACT composite scores**		
Verbal memory, mean [SD]	0.7	0.405
Visual memory, mean [SD]	1.8	0.188
Visual motor speed, mean [SD]	0.2	0.691
Reaction time, mean [SD]	0.3	0.723
**Symptom factors**		
Affective factor, mean [SD]	0.9	0.363
Somatic factor, mean [SD]	0.8	0.386
Sleep factor, mean [SD]	1.5	0.236
Cognitive-migraine-fatigue factor, mean [SD]	0.7	0.400
**VOMS total symptom score, mean [SD]**	1.1	0.284
**Medical History**		
History of prior concussion (Yes/No)	−0.2	0.877
History of migraines (Yes/No)	−2.1	**0.047***
History of motion sickness (Yes/No)	0.2	0.820

Note: * *p*-value was not corrected for multiple comparisons

Table S3. The effect of ImPACT scores, PCSS symptom factors, VOMS, and medical history on RIFG-DAN connectivity in the sample of concussed adolescents without a history of psychiatric disorders or using psychotropic medications (*n* = 31).

Variable	**F[1, 29] or t(30)**	***p*-Value**
**ImPACT composite scores**		
Verbal memory, mean [SD]	0.0	0.944
Visual memory, mean [SD]	1.1	0.307
Visual motor speed, mean [SD]	0.0	0.987
Reaction time, mean [SD]	0.5	0.490
**Symptom factors**		
Affective factor, mean [SD]	0.3	0.615
Somatic factor, mean [SD]	0.2	0.628
Sleep factor, mean [SD]	0.7	0.408
Cognitive-migraine-fatigue factor, mean [SD]	0.4	0.528
**VOMS total symptom score, mean [SD]**	0.0	0.867
**Symptom History**		
History of prior concussion	0.7	0.507
History of migraines	2.0	0.053
History of motion sickness	0.1	0.868
